# Exploring Dose-Dependent Cytotoxicity Profile of *Gracilaria edulis-*Mediated Green Synthesized Silver Nanoparticles against MDA-MB-231 Breast Carcinoma

**DOI:** 10.1155/2022/3863138

**Published:** 2022-02-24

**Authors:** Yugal Kishore Mohanta, Awdhesh Kumar Mishra, Debasis Nayak, Biswajit Patra, Amra Bratovcic, Satya Kumar Avula, Tapan Kumar Mohanta, Kadarkarai Murugan, Muthupandian Saravanan

**Affiliations:** ^1^Department of Applied Biology, School of Biological Sciences, University of Science and Technology Meghalaya, Ri-Bhoi-793101, Meghalaya, India; ^2^Department of Biotechnology, Yeungnam University, Gyeongsan-38541, Gyeongsangbuk-do, Republic of Korea; ^3^Department of Wild Life and Biodiversity Conservation, Maharaja Sriram Chandra Bhanjadeo University, Baripada 757003, India; ^4^School of Life Sciences, Sambalpur University, Odisha, India; ^5^Department of Physical Chemistry and Electrochemistry, Faculty of Technology, University of Tuzla, Univerzitetska 8, 75000 Tuzla, Bosnia and Herzegovina; ^6^Natural and Medical Sciences Research Centre, University of Nizwa, Nizwa 616, Oman; ^7^School of Biological Sciences, University of Science and Technology Meghalaya, Ri-Bhoi-793101, India; ^8^Department of Microbiology, Division of Biomedical Sciences, Mekelle University, Ethiopia; ^9^AMR and Nanotherapeutics Laboratory, Department of Pharmacology, Saveetha Dental College, Saveetha Institute of Medical and Technical Sciences (SIMATS), Chennai, 600077 Chennai, India

## Abstract

Green-based synthesis of metal nanoparticles using marine seaweeds is a rapidly growing technology that is finding a variety of new applications. In the present study, the aqueous extract of a marine seaweed, *Gracilaria edulis*, was employed for the synthesis of metallic nanoparticles without using any reducing and stabilizing chemical agents. The visual color change and validation through UV-Vis spectroscopy provided an initial confirmation regarding the *Gracilaria edulis*-mediated green synthesized silver nanoparticles. The dynamic light scattering studies and high-resolution transmission electron microscopy pictographs exhibited that the synthesized *Gracilaria edulis*-derived silver nanoparticles were roughly spherical in shape having an average size of 62.72 ± 0.25 nm and surface zeta potential of -15.6 ± 6.73 mV. The structural motifs and chemically functional groups associated with the *Gracilaria edulis*-derived silver nanoparticles were observed through X-ray diffraction and attenuated total reflectance Fourier transform infrared spectroscopy. Further, the synthesized nanoparticles were further screened for their antioxidant properties through DPPH, hydroxyl radical, ABTS, and nitric oxide radical scavenging assays. The phycosynthesized nanoparticles exhibited dose-dependent cytotoxicity against MDA-MB-231 breast carcinoma cells having IC_50_ value of 344.27 ± 2.56 *μ*g/mL. Additionally, the nanoparticles also exhibited zone of inhibition against pathogenic strains of *Bacillus licheniformis* (MTCC 7425), *Salmonella typhimurium* (MTCC 3216), *Vibrio cholerae* (MTCC 3904)*, Escherichia coli* (MTCC 1098)*, Staphylococcus epidermidis* (MTCC 3615), and *Shigella dysenteriae* (*MTCC*9543). Hence, this investigation explores the reducing and stabilizing capabilities of marine sea weed *Gracilaria edulis* for synthesizing silver nanoparticles in a cost-effective approach with potential anticancer and antimicrobial activity. The nanoparticles synthesized through green method may be explored for their potential utility in food preservative film industry, biomedical, and pharmaceutical industries.

## 1. Introduction

Nanotechnology is a rapidly growing, dynamic, multidisciplinary research area with potential health, environmental, and socioeconomic applications [1–4]. Natural, engineered, or chemically derived nanoparticles (NPs) are typically ≤100 nm in size and possess unique biophysicochemical properties, such as surface functionalization, an abundant surface-to-volume ratio, target specificity, and controlled release in relative to similar bulk materials. The unique properties of nanoparticles make them suitable for potential applications in cosmetics, biomedicines, and agriculture [5, 6]. Notably, the synthesis of both metal and nonmetal nanoparticles by the use of extracts from both plants and microorganisms has become more prevalent than previous chemical and physical synthesis technologies. The major advantages of green-based synthesis of nanoparticles are (i) lower health and environmental toxicity due to the utilization of natural products during the synthesis of the nanoparticles; (ii) the superior attributes of the green-based nanoparticles that are based on their shape, size, composition, and stability, all of which impact their bioactive properties; (iii) the cost-effectiveness and eco-friendly nature of green-based synthesis; and (iv) and the potential application and acceptability of green-based nanoparticles in food, cosmetics, and textile industries [1–4, 7].

Biofabricated metallic nanoparticles have recently been recognized as a valuable nanomaterial due to their wide range of antimicrobial, antioxidant, and anticancer properties. Metallic nanoparticles (e.g., silver, gold, and platinum) have been utilized in bioelectronics, biosensors, medicine, and pharmaceuticals [8–10]. In particular, silver nanoparticles (AgNPs) have become one of the most commercially important nanoparticles due to their numerous potential applications [9, 11, 12]. Nanoparticles have been explored in the pharmaceutical field for drug delivery, as antibacterial and anticancer drugs, wound dressing, and other applications [13, 14]. Biological synthesis has been researched as a potential platform for the utilization of living organisms, such as plants, microbes, and their primary and secondary metabolites to synthesize nanoparticles [15, 16]. Higher plants, algae, bacteria, fungi, and yeast have been used to synthesize gold (Au), silver (Ag), Palladium (Pd), Platinum (Pt), and selenium (Se) nanoparticles. The derived nanoparticles have been shown to have antimicrobial, anticancer, anthelmintic, and larvicidal activities [11, 17]. The foremost asset of biological synthesis is that it only requires the use of an extract from the host organism that contains chemically active functional groups, a reducing agent, and a capping agent, for the synthesis of nanoparticles, while in chemical synthesis, there is a need for extramural reducing and capping agents [17].

The current trend in the “green synthesis” of nanoparticles is the utilization of algal species, including members of the Chlorophyceae, Phaeophyceae, Cyanophyceae, Rhodophyceae, and Diatoms [18]. This approach to the synthesis of metallic nanoparticles is growing rapidly because (i) it is easy to handle and utilize algae, (ii) algae have a strong ability to accumulate and/or absorb inorganic metallic ions, and (iii) utilization of algae to synthesize nanoparticles represents a natural, ecofriendly, fast, and cost-effective method that has low toxicity [18].

Algae are autotrophic and polyphyletic groups of photosynthetic eukaryotic organisms that are classified as microalgae (unicellular in nature, including diatoms) and multicellular or macroalgae (such as seaweeds). These classifications are generally based on morphological features of algae residing in marine or freshwater habitats, or on the surface of moist rocks [19]. Algae play a key role in aquatic ecosystems; however, some species can form toxic blooms. While nanoparticles have been used to control algal blooms, it should be noted that these blooms represent a valuable biomass source for various deriving compounds that can be utilized in agriculture, pharmaceuticals, cosmetics, bioenergy, etc. [20, 21].

A broad spectrum of natural compounds have been identified in green, red, and brown algae that have a variety of bioactive properties, including antimicrobial, antioxidant, antiviral, anti-inflammatory, cytotoxic, antimitotic, antineoplastic, and antifouling activity [22]. Extracts from marine algae also have the potential to synthesize inorganic metallic nanoparticles [23, 24]. *Gracilaria edulis* is an edible marine alga belonging to the class Gracilariaceae, found exclusively in India. It is a potential warehouse of docosahexaenoic acid (DHA) which is renowned as a vital n-3 polyunsaturated fatty acid (PUFA) [25]. Along with it, the marine alga contains functionally significant amino acids such as aspartic acid, alanine, glutamic acid, and glutamine and chemically important phytochemicals such as polyphenols, phenols, terpenes, steroids, halogenated ketones, fucoxanthin, polyphloroglucinol, and bromophenols [26]. However, the edible marine alga *Gracilaria edulis* has not been explored for their potential in the synthesis of metallic AgNPs. Hence, taking into account the rich phytochemical profile, anticancer efficacy of the crude extract, the current study was designed to explore the efficacy and prospective effect of the green synthesized *Gracilaria edulis*-derived silver nanoparticles (GE-AgNPs) against MDA-MB 231 breast carcinoma cells along with their antibacterial and antioxidant properties.

## 2. Results and Discussion

### 2.1. UV–Vis Spectrum of GE*-*AgNPs

**Figure 1:**
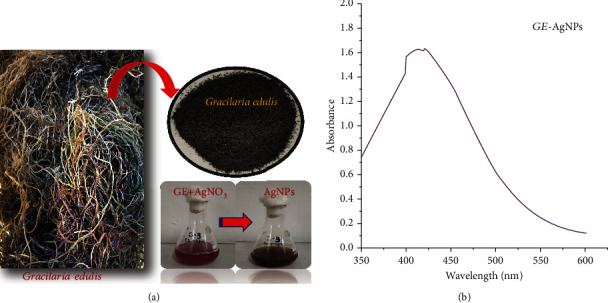
(a) Synthesis of AgNPs from *G. edulis* aqueous extract. (b) UV–Vis spectrophotometric analysis of AgNPs.

The color change inference is considered the preliminary optical inference for the synthesis of AgNPs.  Figure 1(a) shows the color change inference of the primary transparent extract mixture with AgNO_3_ to a reddish-brown solution upon incubation. To confirm the color change inference, the reddish-brown solution obtained was scanned through a UV-Vis spectrophotometer, exhibiting a surface plasmon resonance (SPR) vibration band at 431 nm confirming the synthesis of AgNPs (Figure 1(b)). The results obtained in our study are similar to the previously reported absorption of AgNPs between 410 and 450 nm and accredited to the SPR of AgNPs [27–29]. Notably, use of a UV-Vis spectrophotometer is readily applicable for use in nanoparticle research.

### 2.2. Dynamic Light Scattering (DLS) Analysis of GE-AgNPs

**Figure 2:**
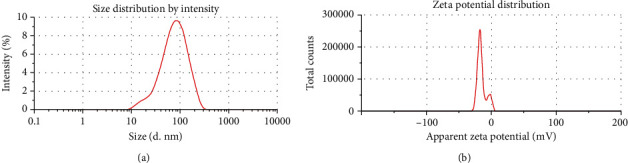
DLS analysis of AgNPs synthesized using *GE* extracts. (a) Average size distribution. (b) Surface charge.

The size and charge are highly related to the potential suitability of a particle in any application. Therefore, it is essential to know the size and charge of a nanoparticle. The size distribution and surface charge of the synthesized GE-AgNPs in an aqueous solution were determined using DLS. The average size of the synthesized GE-AgNPs was calculated to be 62.72 ± 0.25 nm (Figure 2(a)) and the charge was −15.6 ± 6.73 mV (Figure 2(b)). Nanoparticle size plays crucial role in cell transport and communication. Small-sized nanoparticles allow for easier movement of the particles through the plasma membrane of the cell. Therefore, nanosize particles ≤ 100 nm are useful for applications such as drug delivery and construction of biosensors [30–32]. Similarly, the surface charge of GE-AgNPs is also an important attribute as it will affect the ability of the nanoparticles to interact with macromolecules that function in different biochemical pathways [5, 33].

### 2.3. Attenuated Total Reflectance–Fourier Transform Infrared Spectroscopy (ATR-FTIR) Analysis of GE*-*AgNPs

**Figure 3:**
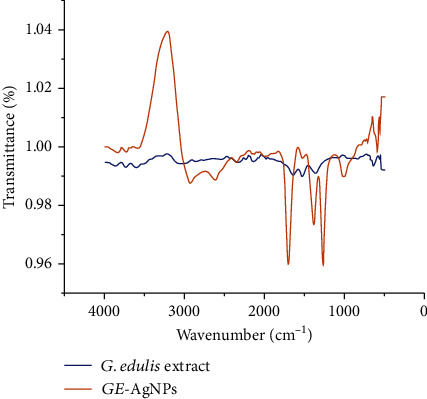
ATR-FTIR analysis of *G. edulis* extract and GE-AgNPs.

The ATR-FTIR spectra of the GE-AgNPs was assessed to determine the functional groups of biomolecules present in the aqueous extract of *G. edulis* that participated in the synthesis and stabilization of the derived AgNPs. Phytochemicals present in the *G. edulis* extract interacted with the nanoparticles during the synthesis process and exhibited sharp peaks at 3228.56, 2933.69, 2625.83, 1714.23, 1393.38, 1278.43, 1034.54, and 661.70 cm^−1^ (Figure 3). It could be seen from Figure 3 that upon interaction with the GE-AgNP extracts, the native vibrational peaks of the inherent AgNPs get interacted with the phenolic groups (-OH) present in the extracts of the biological machinery of the algal species. Such interactions are spectroscopically altered and exhibited an inverse Fourier spectrum at ~3228 cm^−1^. Moreover, besides the –OH functional groups interaction system, there is interionic exchanges taking place at the C-H vibrational forms, where the carbon groups in the biological systems of marine algae extracts gets influenced by the formation of AgNPs formed in the culture at ~2933 cm^−1^. Similarly, the vibrational spectra interactions at the C=O (~1714 cm^−1^), C-O-C, and C-OH (~1278 cm^−1^) indicating the presence of ether-, alcohol-, and sugar-based bond interactions could be easily noticed in the FTIR plots of the *G. edulis* extracts and its out product of *GE*-AgNPs at its corresponding transmittance value. The presence of mild vibration bands at around 660-800 cm^−1^ exhibits the specific fingerprint regions associated with the *G. edulis* extract which signifies the presence of trace elements such as C-I, C-Br which emerges due to the marine nature of the plant extract, and the synthesized GE-AgNPs. Similar results have been reported by various groups for synthesis of AgNPs [34, 35]. It is expected that during the synthesis process of AgNPs from its marine algal source, the biological metabolic groups existing in the algal species like carbon, nitrogen, and oxygen are getting interacted with the differential redox state of the AgNPs produced from its native ionic state to zero valence states of nanoscale particles. Such transformations at the nanoscale phenomenon uncover the explanation of quantum mechanical and redox energetic exchanges taking place at the subatomic stage leading to conversion from ionic state of Ag to its zero state of AgNPs. The biological extracts play hereby a crucial role in the intercalated mechanisms for the lesser toxic product of AgNPs from its ionic precursors, which is much safer and more comprised of lesser defects in its systems as compared to the production of AgNPs from chemical mediated routes.

The overall ATR-FTIR analysis revealed that the proteins and halogenated biomolecules present in the seaweed extract were functioning as both reducing and stabilizing agents during the synthesis of AgNPs, a feature that may be characteristic of all macroalgae extracts. Marine macroalgae are rich sources of both proteins and halogenated compounds that have beneficial applications in many different processes [36, 37]. Notably, the proteins present in the seaweed could bind to the AgNPs via free amine groups, stabilizing clustered nanoparticles through surface-bound proteins [38]. The present results are also strongly supported by the findings presented in previous studies [6, 16, 39].

### 2.4. X-Ray Diffraction (XRD) Analysis of GE*-*AgNPs

**Figure 4:**
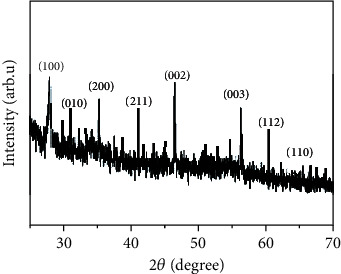
XRD analysis of GE-AgNPs.

XRD is a rapid analytical technique primarily utilized for phase identification of a crystalline material and provides information on unit cell dimensions. Therefore, the analyzed material needs to be finely ground and homogenized to determine its average bulk composition. The results of the XRD analysis of GE-AgNPs are presented in Figure 4. The figure represents a typical XRD diffractogram revealing Bragg peaks predominantly at (angle 2*θ*) at 28.5, 33, 42, and 48.5 (in degree) for the AgNPs synthesized from *G. edulis* seaweed extract which corresponds to (100), (010), (200), and (002), respectively. Miller indices confirm the formation of crystalline elemental AgNPs with a face-centered cubic (FCC) lattice [40, 41]. Thus, the XRD pattern provides strong evidence supporting the UV–Vis spectra and HR-TEM images of the GE-AgNPs.

### 2.5. HR-TEM Analysis of GE*-*AgNPs

**Figure 5:**
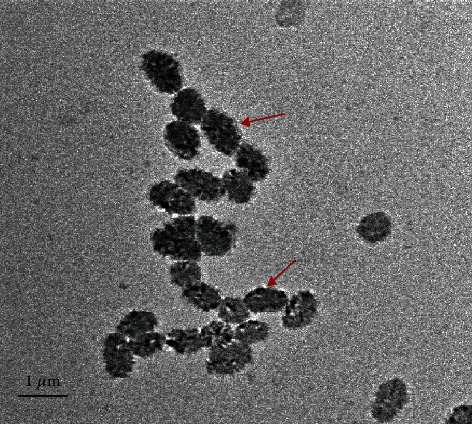
HR-TEM micrograph of GE-AgNPs (indicated by arrows).

HR-TEM micrographs confirmed the spherical shape and polydisperse nature of the GE-AgNPs and their attached biomoieties (Figure 5). The HR-TEM pictographs exhibited that the GE-AgNPs were regular and roughly spherical in shape, with blunt margins. The TEM images also revealed that the nanoparticles were nonagglomerated and freely scattered, making them a strong candidate for biosensor development and drug delivery. The DLS studies also support the properties of the GE-AgNPs revealed in the TEM images, with approximately 80% of the DLS-scanned samples of the GE-AgNPs displaying a size of ~62 nm. Collectively, the dynamic light scattering studies and HR-TEM micrographs confirm that the size of the GE-AgNPs is in the nanorange and that they possess a roughly spherical morphology. This morphological shape and size indicate the potential efficiency of nanoparticles for drug conjugation and drug delivery [42–44].

### 2.6. Qualitative and Quantitative Phytochemical Analyses of the Seaweed Extract

The results of the qualitative and quantitative phytochemical analyses of the aqueous *G. edulis* seaweed extracts are summarized in Tables 1 and 2. The analysis revealed the presence of alkaloids, tannins, phenolic, flavonoids, and saponins, while glycosides, steroids, and sterols were absent. The identified compounds may represent the principal chemical ingredients that are involved in the biosynthesis of AgNPs and define the potential of the nanoparticles for different bioapplications [45–47]. Polyols, terpenoids, phenols, flavones, and polysaccharides have been previously reported to be the principle components in the bio reduction of silver and chloroaurate ions [48]. Importantly, the potential absence of glycosides, steroids, and sterols in the *G. edulis* extracts in our study may be due to the selective qualitative tests that were conducted and/or the extraction procedures. The hypothetical mechanism of the synthesis of AgNPs may involve a cascade of complex antioxidant enzymes [49].

### 2.7. Antibacterial Activity of GE*-*AgNPs

**Figure 6:**
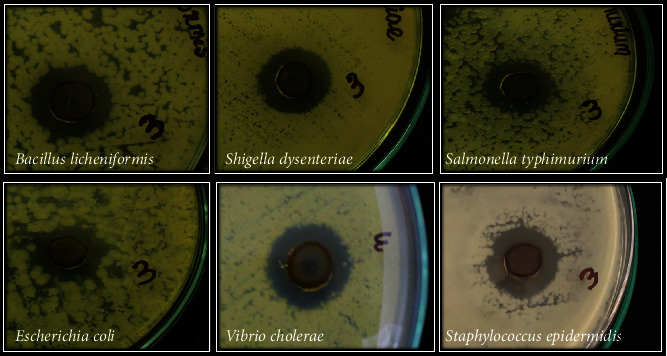
Antibacterial activity (Agar well method) of GE-AgNPs.

**Figure 7:**
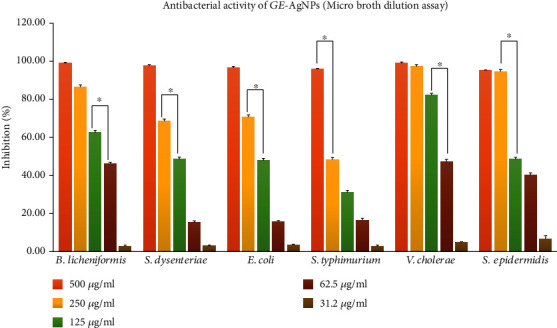
Antibacterial activity (microbroth dilution method) and MICs of GE-AgNPs. Error bar represents standard deviation of mean. ^∗^*p* ≤ 0.05. Significant difference (*p* ≤ 0.05) within a parameter between two lines is denoted by asterisk.

Preliminary evaluation of the antibacterial activity of GE-AgNPs against six pathogenic bacteria was conducted in an agar well diffusion assay (Table 1). Results of this assay indicated that the largest zone of inhibition was observed against *Bacillus licheniformis* and the smallest against *Salmonella typhimurium* (Figure 6). Overall, significant antibacterial activity was observed against *V. cholerae*, *E. coli*, *S. epidermidis*, and *S. dysenteriae.* GE-AgNPs exhibited good bactericidal activity against both Gram-positive and Gram-negative bacteria. A microbroth dilution assay was also used to verify the antibacterial activity of GE-AgNPs and percent to determine percent inhibition and the MICs for each of the pathogenic species of bacteria (Figure 7). The MICs was calculated of six pathogenic bacteria undertaken for the investigation such as *B. licheniformis* (72.84 ± 1.54 *μ*g/mL), *S. dysenteriae* (130.67 ± 2.93 *μ*g/mL), *E. coli* (132.42 ± 3.08 *μ*g/mL), *S. typhimurium* (132.42 ± 3.08 *μ*g/mL), *V. cholerae* (65.58 ± 1.52 *μ*g/mL), and *S. epidermidis* (127.57 ± 3.08 *μ*g/mL). Significant growth inhibition (>94%) was observed in all six pathogenic bacterial species. Although the specific mechanism by which nanoparticles exhibit antibacterial activity is not fully understood, different mechanisms of action have been reported in the literature. Structural changes in the bacterial membrane and ultimate cell death as a result of penetration of nanoparticles into the cell wall due to their anchoring ability have been reported [39, 50, 51]. Enzyme degradation, inactivation of structural proteins, and breakage of genetic materials by AgNPs have also been proposed as mechanisms of action [38, 52, 53]. It has also been suggested that several bacterial cellular enzymes are inactivated by the substantial attachment of Ag ions to –SH groups, a major chemical component of enzyme structure present in the structure [42, 54, 55]. The intermittent interaction of AgNPs with phosphorus and sulfur groups, interfering with the DNA replication processes and dismantling the microbial nuclear system, has also been proposed [56–59]. In our study, the antibacterial results obtained with GE-AgNPs suggests that the seaweed extract-derived AgNPs possess an excellent antimicrobial with widespread potential applications.

### 2.8. Antioxidant Activity of GE-AgNPs

**Figure 8:**
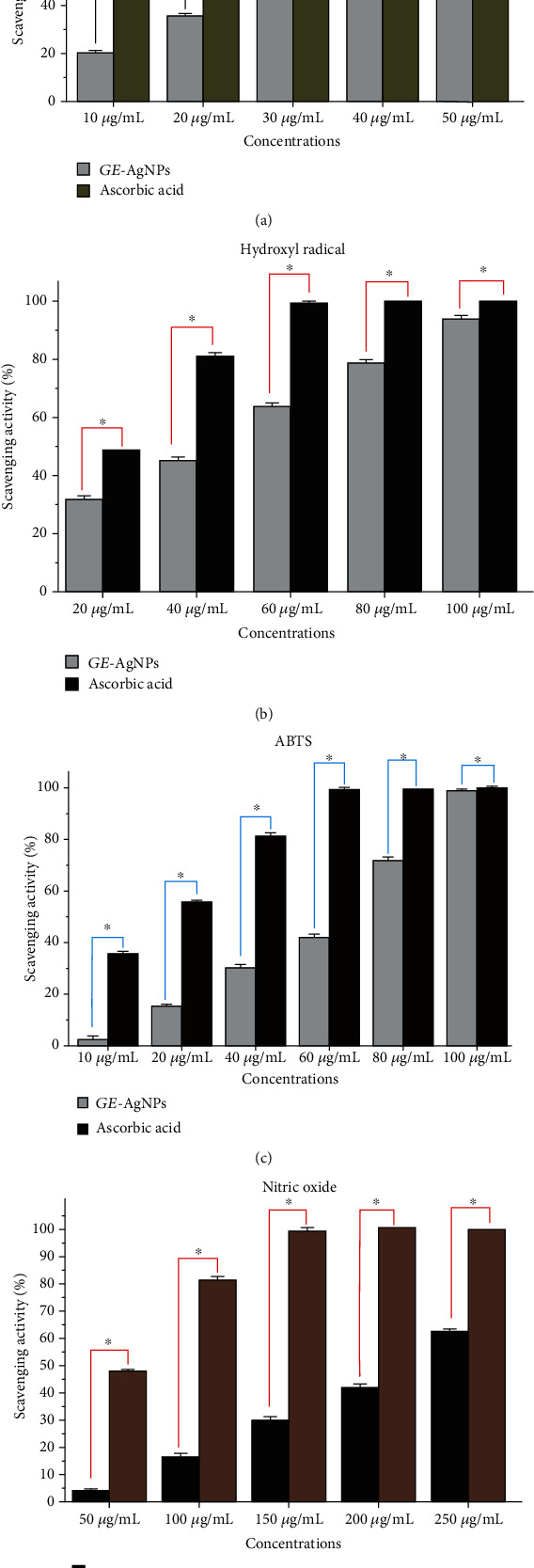
Antioxidant activity of GE-AgNPs in terms of radical scavenging activity: (a) DPPH, (b) hydroxyl ions, (c) ABTS, and (d) nitric oxide radicals. Error bar represents standard deviation of mean. ^∗^*p* ≤ 0.05. Significant difference (*p* ≤ 0.05) within a parameter between two lines is denoted by asterisk.

Abiotic stress induces an overabundance of reactive oxygen species (ROS) that are highly toxic at high levels due to their strong oxidative properties. ROS can damage DNA and RNA, carbohydrates, lipids, and proteins. Chronic oxidative stress can also result in the induction of a variety of different diseases [60], and pharmaceuticals with antioxidant properties have been developed as an option to help minimize oxidative stress in humans. Notably, organisms have evolved an antioxidant system that can scavenge ROS and other free radicals. Delayed and poor absorption potential of exogenous antioxidants, difficulty in passing through cell membranes, and rapid degradation of antioxidants after their delivery, however, represent major challenges to the use of both natural and synthetic antioxidant molecules. Unfortunately, the utilization of nanoparticles as an antioxidant has not been widely recognized and investigated and has been limited to only a few types of nanomaterials [61]. Importantly, functional antioxidant AgNPs derived from the use of various natural extracts obtained from plant species appear to represent a pivotal alternative due to their high stability, biocompatibility, and targeted delivery [6]. Plants, algae, bacteria, and fungi possess a wide array of diverse phenolic compounds and other secondary metabolites that are highly useful as reducing and stabilizing agents. In addition, they also possess excellent antioxidant properties. Therefore, the GE-AgNPs fabricated in the current study were extensively analyzed for their antioxidant properties (Figure 8). The antioxidant activity of *GE*-AgNPs was evaluated using a variety of radical-scavenging assays against different types of reactive radicals, including DPPH, hydroxyl ions, ABTS, and nitric oxide radicals (Figure 8). The DPPH radical-scavenging activity of GE-AgNPs was found to be dose dependent, displaying a maximum inhibition of 86.83% at a concentration of 50 *μ*g/mL. An IC_50_ value of 30.71 ± 0.22 *μ*g/mL was found to be significant, compared to the positive control, ascorbic acid (IC_50_ value 10.33 ± 0.16 *μ*g/mL), thus demonstrating the strong antioxidant property of GE-AgNPs. DPPH is commonly used to evaluate the antioxidant properties of a compound. The radical scavenging activity of GE-AgNPs was also tested against hydroxyl ion (OH^−^) radicals, exhibiting a 94.20% scavenging capacity at 100 *μ*g/mL and an IC_50_ value of 43.85 ± 0.36 *μ*g/mL, compared to the ascorbic acid standard which had an IC_50_ value of 20.43 ± 0.03 *μ*g/mL. Hydroxyl ions readily disrupt disulfide bonds in proteins, resulting in unfolding and refolding into atypical protein structures [62]. Therefore, the current study provides strong evidence for the potential use of GE-AgNPs as antioxidants in biological systems without adverse side effects.

ABTS is another free radical that is generally involved in oxidative damage to cells and polyphenols are capable of minimizing the generation of ABTS. In the present study, the FT-IR analysis of the *G. edulis* extract identified the presence of polyphenols in the synthesized GE-AgNPs. The ABTS scavenging assay indicated 99.16% maximum scavenging activity at 100 *μ*g/mL of GE-AgNPs and an IC_50_ value of 64.77 ± 0.16 *μ*g/mL compared to an IC_50_ value of 16.31 ± 0.11 *μ*g/mL for ascorbic acid. Nitrite has harmful effects on human health due to its reaction with secondary amines in cells, which forms toxic byproducts in human digestive systems [62]. Thus, the activity of GE-AgNPs against nitric oxide radicals was evaluated. A maximum scavenging activity of 62.43% was observed at 250 *μ*g/mL, and the IC_50_ value was determined to be 217.96 ± 1.42 *μ*g/mL; a value that is representative of a moderately active antioxidant compound against nitric oxide radicals. BHT was used as a positive standard in the nitric oxide radical scavenging assay and was determined to have an IC_50_ value of 51.74 ± 0.13 *μ*g/mL. Oxidative stress is believed to play a crucial role in degenerative senescence. As a result, AgNPs with antioxidant capacity could provide a promising therapeutic for the prevention of oxidative stress. *G. edulis* extracts have been previously reported to have antioxidant activity [63]. The present results are also in good accordance to the results obtained from previous studies (Das et al., 2019; Kumar et al., 2020; Otunola and Afolayan, 2018; Ramamurthy et al., 2013b).

### 2.9. Biocompatibility and Cytotoxicity Analyses of GE-AgNPs

**Figure 9:**
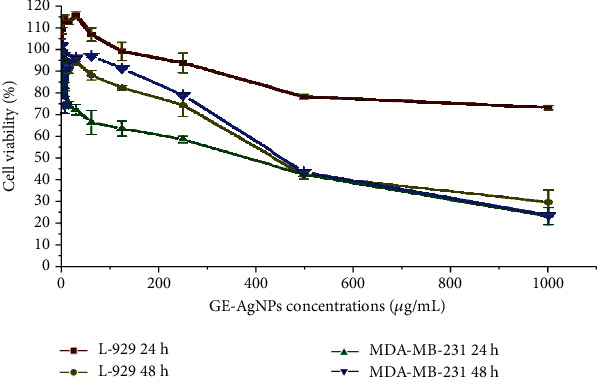
Cell viability of MDA-MB-231 and L-929 after treatment with different concentrations of GE-AgNPs after 24 and 48 h.

A methylthiazolyldiphenyl-tetrazolium bromide (MTT) assay was used to determine cell viability when evaluating the biocompatibility and cytotoxicity of GE-AgNPs. Normal fibroblast cells (L-929) were treated with GE-AgNPs in culture for 24 hour (h) at 37°C and cell viability was subsequently assessed (Figure 9). Results indicated a dose-dependent effect of GE-AgNPs on L-929 cell viability. L-929 cells exposed to a 125 *μ*g/mL concentration of GE-AgNPs exhibited a 99.50% level of cell viability. However, the percent viability gradually reduced as the concentration of GE-AgNPs increased with L-929 cell viability being74.38% when exposed to 1000 *μ*g/mL concentration of GE-AgNPs. These results indicate that GE-AgNPs are relatively nontoxic to normal cells, even at a high concentration. Consequently, these data demonstrate that GE-AgNPs can be potentially used for different biological applications without detrimental effects on the health of cells. The biocompatibility of GE-AgNPs with L-929 cells has been previously reported [64–67].

**Figure 10:**
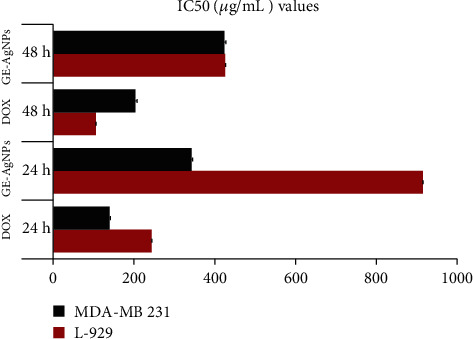
Cell viability (IC_50_ value) of MDA-MB-231, L-929, after treatment with different nanoformulations (Dox: doxorubicin (control), GE-AgNPs) after 24 and 48 h. Error bar represents standard deviation of mean. ^∗^*p* ≤ 0.05. Significant difference (*p* ≤ 0.05) within a parameter between two lines is denoted by asterisk.

In contrast to the biocompatibility of GE-AgNPs with normal fibroblast cells, the viability assay of breast cancer cells (MDA-MB-231) exposed to GE-AgNPs revealed significantly higher levels of cytotoxicity (Figure 10). The breast cancer cells were exposed to ~1000 *μ*g/mL solution of GE-AgNPs for 24 h, which reduced the cell viability to ~21.23%. A time- and dose-dependent cytotoxicity for AgNPs derived from different biological sources against MDA-MB-231 cells has been previously reported [68].

The percentage of viable cancer cells decreased as the concentration of GE-AgNPs increased. A cell viability of 63.81% and 94.06% was observed after exposure to a 125 *μ*g/mL and 1.95 *μ*g/mL solution of GE-AgNPs, respectively. The MIC of *GE*-AgNPs was calculated as 344.27 ± 2.56 *μ*g/mL against MDA-MB-231. These results indicate that the MDA-MB-231 cell lines exhibit a concentration-dependent response with response to viability. The level of cytotoxicity, however, did not appear to be time dependent as an identical percentage of viable cells was observed after both 24 and 48 h exposure to the same concentration of GE-AgNPs. The IC_50_ values for doxorubicin (used as a positive control) were higher in MDA-MB-231 cells than in L-929 cells after 24 hr or 48 hr of exposure. In contrast, the IC_50_ values for GE-AgNP treatment were significantly lower in MDA-MB-231 cells than in L-929 cells after exposure for 24 or 48 h (Figure 10).

AgNPs induce cytotoxic effect due to their impact on different metabolic pathways. Another study reported that the cytotoxicity of AgNPs results from an increase in ROS production [69]. Previous studies stated that the introduction of AgNPs into target cells could promote the overproduction of intracellular ROS, which activates apoptosis-associated metabolic pathways including p53, MAPK, and AKT apoptotic signaling pathways [70, 71]. Similar to other metal nanoparticles, AgNPs also promote oxidative stress in cells by inducing the overproduction of ROS [70]. Mitochondria are vital sources of apoptosis signals and the effect of Ag-NPs on mitochondrial membrane permeability results in the loss of mitochondrial membrane integrity, leading to caspase-dependent apoptotic cell death [72]. In addition to AgNPs stimulating apoptosis in cells, it is more than likely that future studies will reveal other mechanisms by which AgNPs establish their cytotoxicity.

## 3. Materials and Methods

### 3.1. Collection and Preparation of Seaweed Extract

The red seaweed, *G. edulis* (Linnaeus), was collected from Chilika Lake, Odisha, India (19° 43′ 0^″^ N, 85° 19′ 0^″^ E) and transported to a laboratory in a portable ice cooler. The harvested seaweed was then thoroughly cleaned in running tap water followed by distilled water to remove extraneous materials and to substantially reduce the salt content, after which the seaweed was dried in a shady, open-air environment for 3–5 days. The dried seaweed was subsequently ground to a fine powder using a commercial-grade mixer grinder. Then, 5.0 g of the *G. edulis* seaweed powder was boiled in 50 mL of sterilized Milli Q water for 20-30 min and subsequently filtered through Whatman No. 1 filter paper. The filtered extract was stored at -4°C until further use.

### 3.2. Synthesis of GE-AgNPs

A total of 10 mL of seaweed extract was mixed with 90 mL of a 1.0 mM aqueous solution of AgNO_3_ [45, 73] and incubated at room temperature on a rotary shaker for 1 hr. A color change in the reaction solution from light brick red to deep brown was noted by visual observation and was used to confirm the completion of the AgNP synthesis. The synthesized AgNPs were pelleted by centrifugation at 8000 rpm for 15 min at 10°C. The obtained AgNPs were dried and stored at 4°C for characterization and assessment of their bioactive properties.

### 3.3. Characterization of GE-AgNPs

The synthesis of the AgNPs using an aqueous extract of *G. edulis* was periodically monitored by UV-Vis spectrophotometer (Lambda 35R PerkinElmer, USA) in the range of 350-600 nm. The UV-visible spectra of the synthesis reaction solution were recorded as a function of reaction time at a resolution of 1 nm at 25°C. The surface charge and average size of the AgNPs were analyzed using a Zetasizer (ZS 90, Malvern, UK). The purified nanoparticle samples were diluted tenfold in PBS (0.15 M, pH 7.2). Aliquots were sampled and placed in dynamic light scattering (DLS) cuvettes and then evaluated for equivalent size distribution, diameters, and zeta potential. Particle diameters were assessed at a scattering angle of 90° at 25°C. ATR-FTIR spectroscopy analysis of the, *G. edulis* aqueous extract and the synthesized *GE*-AgNPs was conducted to substantiate the potential role of the various functional chemical groups present in the seaweed extracts on the modification of the surface of the synthesized nanoparticles. ATR-FTIR was conducted on a Bruker ALPHA spectrophotometer (Ettlinger, Germany) at a resolution of 4 cm^−1^. The samples were evaluated in the spectral region of 4000 to 500 cm^−1^ by taking an average of 25 scans per sample. For continuous observations, one drop of the sample was kept on the sample holder and the samples were scanned and the obtained results were analyzed using OPUS software. The crystalline properties of the AgNPs were assessed using an X-ray diffractometer (PANalytical X'Pert, Almelo, The Netherlands) equipped with a Ni filter and a CuK (*l* = 1.54056 Å) radiation source. The scanning rate was 0.05°, while the diffraction angle varied from 20–80°. High-resolution transmission electron microscopy (Technai™ F30 G2 STWIN, FEI, Lincoln, NE, USA) was used to observe the nanomorphology of the AgNPs. The synthesized AgNPs were placed on a coated copper grid with a 300 mesh size and observed at an accelerating voltage of 300 kV.

### 3.4. Qualitative and Quantitative Analyses of the Seaweed Extract

The qualitative phytochemical analysis of the *G. edulis* extract was performed following standard methods [63]. The obtained results were qualitatively expressed as positive (+ve) or negative (-ve). The chemicals and reagents used for the study were purchased from Sigma–Aldrich (India).

#### 3.4.1. Total Phenol Content

Total phenol content (TPC) in the seaweed extract was estimated using the Folin–Ciocalteu method with slight modifications as described by Lim et al. (2007). The analysis was performed in triplicate. TPC was expressed as gallic acid equivalents (GAE) in mg/g sample.

The concentration of total phenolic compounds in the extract was determined using the following formula:

(1) $$\begin{array}{c} T=C*\frac{V}{M}, \end{array}$$

where *T* is the total phenolic content mg/gm of seaweeds extract in GAE, *C* is the concentration of Gallic acid from the calibration curve in mg/mL, *V* is the volume of the extract in mL, and *M* is the Wt of the seaweeds extracts in g.

#### 3.4.2. Total Flavonoid Content

Total flavonoid content (TFC) of the *G. edulis* extract was determined using an aluminum chloride (AlCl_3_) colorimetric assay and expressed as milligrams of quercetin equivalents per gram dry mass (mg·Q/g dw) [74]. The analysis was performed in triplicate.

### 3.5. Antibacterial Activity of the GE-AgNPs

#### 3.5.1. Bacterial Strains

The six species of human pathogenic bacteria, *Bacillus licheniformis* (MTCC 7425), *Salmonella typhimurium* (MTCC 3216), *Vibrio cholerae* (MTCC 3904), *Escherichia coli* (MTCC 1098), *Staphylococcus epidermidis* (MTCC 3615), and *Shigella dysenteriae* (MTCC-9543) were used in the antibacterial assay. The bacterial strains were purchased from MTCC, Pune.

#### 3.5.2. Agar Well Diffusion and Microbroth Dilution Methods

A small colony of each targeted bacterial strain was inoculated from a stock agar slant into 2 mL Muller Hinton (MH) broth medium (0.015% soluble starch, 0.2% beef extract, and 1.75% casamino acids) under proper aseptic conditions. The inoculated tubes were incubated overnight at 37°C on a rotary shaker at 200 rpm.

The assessment of the antibacterial activity of the synthesized GE-AgNPs against the selected pathogenic bacteria was conducted using a well diffusion assay with Muller Hinton Agar (MHA). Briefly, 100 *μ*L of each bacterium was seeded over the prepared MHA plates. Test wells (5 mm diameter and 3 mm deep) were then made in the inoculated agar medium using a sterile cork borer. Each well was then filled with 50 *μ*L of GE-AgNPs. Wells filled with 50 *μ*L of silver nitrate (AgNO_3_) solution served as the control while wells filled with the antibiotic, gentamicin, were used as a positive control. The inoculated plates were kept in an incubator at 37°C for 24 h. Following the period of incubation, the diameter of inhibition zones was measured and a zone diameter ≥ 8 mm was recorded as a positive antibacterial activity.

Antibacterial assessment was carried out using the microbroth dilution method. The minimum inhibitory concentration (MIC) of the GE-AgNPs on bacterial strains was also assessed [75]. Inhibition ≥ 90% in the microbroth dilution assay was used as an indication of good antibacterial activity, and additional experiments were carried out for MIC estimation. The test inoculum (190 *μ*L; *A*_600_ = 0.1) were incubated in 10 *μ*L of different concentrations (500-31.25 mg/mL; twofold dilution) of the GE-AgNPs until the level of inhibition was found to be <50%. The assays were conducted in 96-well plates, and microbial growth was determined in a microplate reader (Bio-Rad, USA) at 600 nm. The numerical MIC values were calculated using IC_50_/IC_90_ Laboratory Excel Calculation formulas and expressed as IC_50_. All of the assays were conducted in triplicate, and zones of inhibition were expressed in a mean ± SD.

### 3.6. Antioxidant Activity of the GE-AgNPs

The antioxidant activity of the GE-AgNPs was assessed by its radical scavenging ability.

#### 3.6.1. DPPH Radical Scavenging Activity

The radical scavenging activity of GE-AgNPs was determined using the 1,1-diphenyl-2-picryl-hydrazil (DPPH) assay with slight modification (Arul Kumar et al., 2018; Lim et al., 2007). Different concentrations (10, 20, 30, 40, and 50 *μ*g/mL) of GE-AgNPs were used in the assay. Ascorbic acid in equivalent concentrations was used as a positive control, and results were expressed as percentage (%) radical scavenging activity. The MIC for DPPH radical scavenging activity was also calculated and expressed as an IC_50_.

#### 3.6.2. Hydroxyl Radical Scavenging Activity

Hydroxyl (OH^−^) radical scavenging activity of GE-AgNPs was evaluated as previously described [76] using different concentrations (20, 40, 60, 80, and 100 *μ*g/mL) of GE-AgNPs. Ascorbic acid at equivalent concentrations was used as a positive control. The MIC for hydroxyl radical scavenging activity was also calculated and expressed as an IC_50_.

#### 3.6.3. 2,2-Azino-bis(3-ethylbenzothiozoline-6-sulfonic acid) Diammonium Salt (ABTS) Radical Scavenging Activity

ABTS radical scavenging activity of the GE-AgNPs was also determined using a radical cation decolorization assay as previously described [77] using different concentrations (20, 40, 60, and 80 *μ*g/mL) of GE-AgNPs. Ascorbic acid at equivalent concentrations was used as a positive control. The MIC for ABTS radical scavenging activity was also calculated and expressed as an IC_50_.

#### 3.6.4. Nitric Oxide Radical (NO^∗^) Scavenging Activity

Nitric oxide radical scavenging activity of the GE-AgNPs was evaluated using the method described by Garrat (1964) with slight modification. Briefly, sodium nitroprusside (Na_2_[**Fe** (**CN**)_5_NO]2H_2_O) in aqueous solution generates nitric oxide spontaneously at physiological pH, which immediately interacts with oxygen to produce nitrite ions (NO^2-^), which can be determined by the Griess-Ilosvay reaction. A standard method [63] to evaluate the NO^∗^ scavenging activity was also used. Different concentrations (50, 100, 150, 200, and 250 *μ*g/mL) of GE-AgNPs were used in the assay, and BHT was used as a positive control. The MIC for NO^2-^ radical scavenging activity was also calculated and expressed as an IC_50_.

### 3.7. Biocompatibility and Cytotoxicity Analysis of GE-AgNPs

#### 3.7.1. Cell Culture

A normal fibroblast cell line (L-929) and a breast cancer cell line (MDA-MB 231) were used in the biocompatibility and cytotoxicity assays. Both cell lines were seeded on Dulbecco's modified Eagle's medium and M-199 medium supplemented with 10% fetal bovine serum (FBS), as well as streptomycin sulfate and benzyl antibiotics at a final concentration of 100 *μ*g/mL and 100 U/mL, respectively. Cell cultures were incubated at 37°C (5% CO_2_) for 24 h, for the duration of the assays. The cells were trypsinized using 0.25% Trypsin-EDTA at a 70 to 80% confluence. Cells were counted and then placed in a 96-well enzyme-linked immunosorbent assay (ELISA) plate at a density of 5 × 10^3^ cells/well to conduct MTT assay. All cell culture chemicals are purchased from Sigma–Aldrich (India).

#### 3.7.2. MTT Assay

Biocompatibility and cytotoxicity were evaluated using a MTT colorimetric assay after 24 and 48 h. incubation of the cell lines with the GE-AgNPs. When the cells were at 90% confluency, the media was removed and the cells were exposed with fresh medium containing different concentrations of GE-AgNPs (viz., 100, 200, 400, 600, 800, and 1000 *μ*g/mL) and was further incubated for 24 h. Doxorubicin was employed as a positive control. Similar to the GE-AgNPs, various concentrations of DOX was used to see its efficacy along with the synthesized GE-AgNPs. A stock solution of MTT (1 mg/mL) in PBS was prepared immediately prior to use. A 500 *μ*L volume of the MTT solution (50 *μ*g/mL MTT in the culture medium) was added to each culture dish and left uncovered. Cells were incubated for 3 hr, after which the reduced formazan was extracted with 500 *μ*L of DMSO and absorbance was measured at 595 nm in a microtiter plate reader (Bio-Rad, USA). Cell viability was assessed as the percentage absorption of treated cells relative to the untreated and control cells.

### 3.8. Statistical Analysis

All assays in this study were performed in triplicate. The results of the antioxidant assays are presented as a percentage inhibition, while the cytotoxicity results are presented as % viability, relative to the control. The antioxidant and cytotoxicity assay data for the different treatment groups vs. the controls were statistically evaluated using Student's *t*-test (*p* ≤ 0.05).

## 4. Conclusion

Marine macroalgae or sea weed *G. edulis* has momentous attributes in the green synthesis process of metal nanoparticles, like other biological resources such as plant, bacteria, fungi, macrofungi or mushrooms, and yeast. Due to the encouraging involvement of algae in the nanotechnology advancement, the separate branch known as phyconanotechnology is growing enormously to substantiate the different biomedical, agriculture, and environmental issues. Various studies on the biosynthesis of nanoparticles using seaweed extracts have been conducted. In the current investigations, the physiochemical characterization of the synthesized AgNPs demonstrated the stable synthesis of AgNPs that can potentially be used in different applications. The antibacterial, antioxidant, biocompatibility, and cytotoxicity of the AgNPs indicate their potential commercial utility in biomedical and pharmaceutical industries. The use of seaweed extract in nanoparticle biosynthesis is highly advantageous due to the presence of a variety of secondary metabolites in the extract that affect the properties of the synthesized nanoparticles and exhibit low cytotoxicity to healthy cells. The use of “green-based” synthesis of nanoparticles is compatible with large-scale production and smooth downstream processing. Further studies are warranted and necessary to explore the use of seaweed extracts in nanotechnological applications and to fully understand the properties of seaweed-fabricated metal nanoparticles, their mechanism of action, and their potential applications in food, health, and environmental industries. Comprehensively, the nanobiotechnology that utilizes the sources from algae and blue-green algae to synthesize nanomaterials is in the budding stage, and further research and development are necessary.

## Figures and Tables

**Table 1:** Qualitative phytochemical analysis of an aqueous extract of *G*. *edulis* seaweed.

Phytoconstituent	Observation
Alkaloids	+
Tannins and phenolic compounds	+
Glycoside	-
Flavonoids	+
Steroids and sterols	-
Triterpenoids	+
Saponins	+

Notes: +: present; -: absent.

**Table 2:** Quantitative analysis of total phenolics and carbohydrates in an aqueous extract of *G*. *edulis* seaweed.

Phytochemical constituent	mg/g dry weight (mean ± SD)
TPC	7.27 ± 1.10
TFC	4.70 ± 0.63

## Data Availability

The data supporting the reported results are available upon request from the first and corresponding author.

## Additional Points

*Research Highlights:* (1) Green synthesis of silver nanoparticles from marine sea weed *Gracilaria edulis* for efficient use in biomedical industry sector is highly demanding for its ecofriendly and less toxic byproduct production, (2) The use of marine sea weed for such green synthesis is highly attractive to a combinatorial approach in addition of natural compounds with nanoparticles, (3) Silver nanoparticles also exhibited potential antibacterial activity against *Bacillus licheniformis*, *Shigella dysenteriae*, *Escherichia coli*, *Salmonella typhimurium*, *Vibrio cholerae*, and *Staphylococcus epidermidis,* (4) AgNPs performed high efficacy of DPPH, hydroxyl radical, ABTS, and nitric oxide radical scavenging activity, (5) AgNPs are highly biocompatible to normal fibroblast cell line L-929 and cytotoxic against breast cancer (MDA-MB-231) cells
